# Risk of metachronous colorectal cancer after surgical resection of index rectal cancer in Lynch syndrome: a multicenter retrospective study in Japan

**DOI:** 10.1007/s00595-024-02815-z

**Published:** 2024-03-19

**Authors:** Kenichi Chikatani, Hideyuki Ishida, Yoshiko Mori, Takeshi Nakajima, Arisa Ueki, Kiwamu Akagi, Akinari Takao, Masayoshi Yamada, Fumitaka Taniguchi, Koji Komori, Kazuhito Sasaki, Tomoya Sudo, Yasuyuki Miyakura, Akiko Chino, Tatsuro Yamaguchi, Kohji Tanakaya, Naohiro Tomita, Yoichi Ajioka

**Affiliations:** 1 Department of Digestive Tract and General Surgery, Saitama Medical Center, Saitama Medical University, 1981 Kamoda, Kawagoe, Saitama 350-8550 Japan; 2 The Committee of Hereditary Colorectal Cancer in the Japanese Society for Cancer of the Colon and Rectum (JSCCR), Tokyo, Japan; 3 Department of Clinical Genetics, Cancer Institute Hospital, Japanese Foundation for Cancer Research, Tokyo, Japan; 4 Department of Molecular Diagnosis and Cancer Prevention, Saitama Cancer Center, Saitama, Japan; 5 Department of Gastroenterology, Tokyo Metropolitan Cancer and Infectious Diseases Center, Komagome Hospital, Tokyo, Japan; 6 Endoscopy Division, National Cancer Center Hospital, Tokyo, Japan; 7 Department of Surgery, National Hospital Organization Iwakuni Clinical Center, Yamaguchi, Japan; 8 Department of Gastroenterological Surgery, Aichi Cancer Center Hospital, Aichi, Japan; 9 Department of Surgical Oncology, Faculty of Medicine, The University of Tokyo, Tokyo, Japan; 10 Department of Surgery, Kurume University, Fukuoka, Japan; 11 Department of Surgery, Saitama Medical Center, Jichi Medical University, Saitama, Japan; 12 Department of Gastroenterology, Cancer Institute Hospital, Japanese Foundation for Cancer Research, Tokyo, Japan; 13 Department of Clinical Genetics, Tokyo Metropolitan Cancer and Infectious Diseases Center, Komagome Hospital, Tokyo, Japan; 14 Cancer Treatment Center, Toyonaka Municipal Hospital, Osaka, Japan; 15 Japanese Society for Cancer of the Colon and Rectum, Tokyo, Japan; 16 Division of Molecular and Diagnostic Pathology, Graduate School of Medical and Dental Sciences, Niigata University, Niigata, Japan; 17 Present Address: Department of Medical Ethics and Medical Genetics, Kyoto University School of Public Health, Kyoto, Japan

**Keywords:** Lynch syndrome, Rectal cancer, Colorectal cancer, Metachronous colorectal cancer

## Abstract

**Purpose:**

This study evaluated the risk of metachronous colorectal cancer (CRC) after resection of index (first) rectal cancer in patients with Lynch syndrome (LS).

**Methods:**

Clinicopathological data of patients with genetically proven LS were retrospectively analyzed in this multicenter Japanese study. The cumulative incidence of metachronous CRC and the overall survival were compared between patients with index rectal cancer (rectal group) and those with index colon cancer (colon group).

**Results:**

The median age at index CRC surgery was lower in the rectal group than in the colon group (37 vs. 46 years old, *P* = 0.01). The cumulative 5-, 10-, and 20-year incidences of metachronous CRC were 3.5%, 13.9%, and 21.1%, respectively, in the rectal cancer group and 14.9%, 22.0%, and 57.9%, respectively, in the colon cancer group (*P* = 0.02). The overall survival curves were not significantly different between two groups (*P* = 0.23).

**Conclusion:**

This is the first report from an East Asian country to report the risk of metachronous CRC after resection of index rectal cancer in patients with LS. Despite this study having several limitations, we cannot recommend extended resection, such as total proctocolectomy, for index rectal cancer as a standard surgical treatment in patients with LS.

## Introduction

Lynch syndrome (LS) is an autosomal dominant inherited cancer susceptibility syndrome caused by germline pathogenic variants, such as DNA mismatch repair genes (*MLH1*, *MSH2*, *MSH6*, and *PMS2*) or germline deletion of the 3′ site of *EPCAM*, which is upstream of *MSH2*. It is characterized by a high risk of multiple synchronous and metachronous colorectal cancers. The cumulative risk of colorectal cancer (CRC) in carriers of MMR gene variants depends on the causative gene and has been reported to be 10–46% [1, 2].

In terms of reducing the risk of metachronous CRC after surgery for index (first) colon cancer (CC) in MMR gene variant carriers, the American Gastroenterological Association guidelines recommend extended surgery, such as total colectomy (TC) with ileosigmoidal/ileorectal anastomosis (IRA) [3]. However, rectal cancer (RC) accounts for only 12.7%-18.0% of index CRC in MMR gene variant carriers [1, 2, 4, 5], and there are very limited data on the risk of metachronous CRC and the site of occurrence after index RC surgery. Therefore, the choice of surgical procedure for index RC in MMR gene variant carriers—specifically, whether anterior resection/abdominoperineal resection is commonly performed for sporadic (non-hereditary) RC or extended surgery such as total proctocolectomy (TPC) with ileal pouch-anal anastomosis (IPAA)—remains controversial.

The present study addressed these issues and compared the incidence of cumulative metachronous CRC development, the site of metachronous CRC occurrence, and the overall survival (OS) between patients with genetically proven LS who developed index RC and those who developed index CC. This study was conducted as part of a nationwide Japanese multicenter study conducted by the Committee of Hereditary Colorectal Cancer of the Japanese Society for Cancer of the Colon and Rectum (JSCCR).

## Methods

### Study sample

This nationwide Japanese multicenter study conducted by the Committee of Hereditary Colorectal Cancer of the JSCCR (No. 90-7) was approved by the ethics committees of the authors' institution. We retrospectively enrolled 316 patients with genetically confirmed LS in this study. Only CRC patients who underwent open or laparoscopic resection of colorectal adenocarcinoma (including intramucosal carcinoma) were eligible. Seventy-nine patients who did not undergo surgery and 22 without clinicopathological data were excluded. Nine patients who underwent total colectomy (TC) as the initial surgery, 11 who underwent multi-segmental colectomy for synchronous CRC, and 2 with appendiceal carcinoma were excluded. A total of 193 patients were thus included in this study.

The following data were collected: age, sex, causative gene type, proband or relative status, diagnosis of LS before surgery, tumor location, surgical procedure, tumor stage, tumor histology, and endoscopic surveillance. Tumors were classified according to the Tumor-Node-Metastasis (TNM) Union for International Cancer Control staging system [6].

Patients who underwent colectomy for index CC (cecum – rectosigmoid colon) comprised the colon group, whereas those who underwent low anterior resection and abdominoperineal resection of index RC comprised the rectal group. The right-sided colon was defined from the cecum to the oral side of the splenic flexure, and the left-sided colon was defined from the anal side of the splenic flexure to the rectosigmoid colon.

In accordance with the criteria of the Japanese Classification of Colorectal, Appendiceal, and Anal Carcinoma (the 3rd English Edition) and the Surveillance Epidemiology and End Results Programme (SEER), “metachronous” was defined as tumors diagnosed two months or more after surgery for index CRC [7, 8].

### Statistical analysis

The chi-square test or Fisher’s exact probability test was used to compare categorical variables, and the Mann–Whitney U test was used to compare continuous variables between groups. The cumulative incidences of metachronous CRC and the OS were determined using the Kaplan–Meier method, and the log-rank test was used to compare the curves between the groups. The time at risk was calculated from the day of the index CRC surgery to the day of the initiation of metachronous CRC treatment. When multiple metachronous CRC were present, the date of treatment for the earliest metachronous CRC was used as the endpoint. In patients without metachronous CRC, the date of death or the last follow-up, whichever occurred first, was used as the endpoint. Metachronous CRC events were counted only once, and subsequent CRC events were not considered.

All statistical tests were two-tailed, and *P* < 0.05. The statistical software program JMP Pro 16.1.0 (SAS Institute Inc., Cary, NC, USA) was used for statistical analyses.

## Results

### Patient characteristics

Table 1 shows the baseline characteristics of the 193 eligible patients. The median age at the time of index CRC surgery was 45 (range 14–80) years old. The median follow-up time was 96 (range 4–534) months; 89 patients (46.1%) were female, and 174 patients (90.2%) were probands. Seven patients (3.6%) were genetically diagnosed with LS before surgery for CRC. The causative genes were as follows: *MLH1* in 81 patients (42.0%), *MSH2* in 83 patients (43.0%), *MSH6* in 17 patients (8.8%), *PMS2* in 10 patients (5.2%), and *EPCAM* in 2 patients (1.0%).

**Table 1:** Patient characteristics

	Total (n = 193)	Colon group (n = 162)	Rectal group (n = 31)	*P* value
Follow-up time				
Median months (range)	96 (4–534)	96 (4–534)	105 (8–345)	0.42
Age at surgery for index colorectal cancer				
Years (range)	45 (14–80)	46 (14–80)	37 (14–67)	0.01
Sex				
Female (%)	89 (46.1%)	78 (48.2%)	11 (35.5%)	0.24
Causative gene				
* MLH1*	81 (42.0%)	71 (43.8%)	10 (32.3%)	0.74
*MSH2*	83 (43.0%)	67 (41.4%)	16 (51.6%)	
*MSH6*	17 (8.8%)	14 (8.7%)	3 (9.7%)	
*PMS2*	10 (5.2%)	8 (4.9%)	2 (6.4%)	
*EPCAM*	2 (1.0%)	2 (1.2%)	0 (0%)	
Proband or relative				
Proband	174 (90.2%)	145 (89.5%)	29 (93.6%)	0.74
A diagnosis of Lynch syndrome				
Prior to surgery	7 (3.6%)	7 (4.3%)	0 (0%)	0.60
Tumor location				
Cecum	32 (16.6%)	32 (19.8%)	–	–
Ascending colon	39 (20.2%)	39 (24.1%)	–	
Transverse colon	37 (19.2%)	37 (22.8%)	–	
Descending colon	11 (5.7%)	11 (6.8%)	–	
Sigmoid colon	37 (19.2%)	37 (22.8%)	–	
Rectosigmoid colon	6 (3.1%)	6 (3.7%)	–	
Rectum	31 (16.0%)	–	31 (100.0%)	
Surgical procedure				
Ileocecal resection	24 (12.4%)	24 (14.8%)	–	–
Right hemicolectomy	64 (33.2%)	64 (39.5%)	–	
Transverse colectomy	16 (8.3%)	16 (9.9%)	–	
Descending colectomy	3 (1.5%)	3 (1.9%)	–	
Sigmoidectomy	37 (19.2%)	37 (22.8%)	–	
Left hemicolectomy	12 (6.2%)	12 (7.4%)	–	
High anterior resection	6 (3.1%)	6 (3.7%)	–	
Low anterior resection	27 (14.0%)	–	27 (87.1%)	
Abdominoperineal resection	4 (2.1%)	–	4 (12.9%)	
Stage				
0	7 (3.6%)	6 (3.7%)	1 (3.2%)	0.19
I	40 (20.7%)	29 (17.9%)	11 (35.5%)	
II	62 (32.1%)	56 (34.6%)	6 (19.4%)	
III	47 (24.4%)	38 (23.5%)	9 (29.0%)	
IV	4 (2.1%)	3 (1.8%)	1 (3.2%)	
Unknown	33 (17.1%)	30 (18.5%)	3 (9.7%)	
Histology				
Well-/moderated differentiated	122 (63.2%)	98 (60.5%)	24 (77.4%)	0.18
Poorly differentiated/Mucinous	33 (17.1%)	29 (17.9%)	4 (12.9%)	
Unknown	38 (19.7%)	35 (21.6%)	3 (9.7%)	
Colonoscopy surveillance after surgery				
Within 3 years	106 (54.9%)	87 (53.7%)	19 (61.3%)	0.43

The rectal group comprised 31 patients (16.1%), whereas the colon group comprised 162 patients (83.9%). Significant differences were found in the median age at surgery for the index CRC between the rectal (37 [range 14–67] years old) and colon (46 [range 14–80] years old) groups (*P* = 0.01).

However, no significant differences were found between the two groups in terms of clinical and pathological factors, such as follow-up time, sex, causative gene, proband or relative status, timing of the LS diagnosis, stage, histology, and colonoscopy surveillance performed within three years after surgery for the index CRC.

### Sites of metachronous CRC

The sites of metachronous CRC, according to the index cancer, are shown in Table 2. Among the 31 patients in the rectal group, metachronous CRC was identified in 6 (19.4%), including in the right-sided colon in 5 (83.3%) and left-sided colon in 1 (16.7%); none had it in the remnant rectum. Among the 20 male patients in this group, 6 (30%) had metachronous CRC, whereas no metachronous CRC was observed in female patients. Among the 162 patients in the colon group, metachronous CRC was detected in 61 (37.7%), including in the right-sided colon in 35 (57.4%), left-sided colon in 18 (29.5%), and rectum in 8 (13.1%). Among the 84 male patients in this group, 32 (38.1%) had metachronous CRC; among the 78 female patients, 29 (37.2%) had metachronous CRC.

**Table 2:** Site of metachronous colorectal cancer after surgery for index colorectal cancer

Tumor location	Colon group (n = 61)			Rectal group (n = 6)		
	All (n = 61)	Males (n = 32)	Females (n = 29)	All (n = 6)	Males (n = 6)	Females (n = 0)
Right-sided colon	35 (57.4%)	17 (53.1%)	18 (62.1%)	5 (83.3%)	5 (83.3%)	0 (0%)
Cecum	3 (4.9%)	1 (3.1%)	2 (6.9%)	2 (33.3%)	2 (33.3%)	0 (0%)
Ascending colon	17 (27.9%)	9 (28.1%)	8 (27.6%)	2 (33.3%)	2 (33.3%)	0 (0%)
Transverse colon	15* (24.6%)	7 (21.9%)	8 (27.6%)	1 (16.7%)	1 (16.7%)	0 (0%)
Left-sided colon	18 (29.5%)	11 (34.4%)	7 (24.1%)	1 (16.7%)	1(16.7%)	0 (0%)
Descending colon	5 (8.2%)	2 (6.3%)	3 (10.3%)	1 (16.7%)	1 (16.7%)	0 (0%)
Sigmoid colon	13 (21.3%)	9 (28.1%)	4 (13.8%)	0 (0%)	0 (0%)	0 (0%)
Rectosigmoid colon	0 (0%)	0 (0%)	0 (0%)	0 (0%)	0 (0%)	0 (0%)
Rectum	8 (13.1%)	4 (12.5%)	4 (13.8%)	0 (0%)	0 (0%)	0 (0%)

*Including a case of synchronous advanced transverse colon and sigmoid intramucosal colon cancer

### Cumulative risk of metachronous CRC

**Fig. 1:**
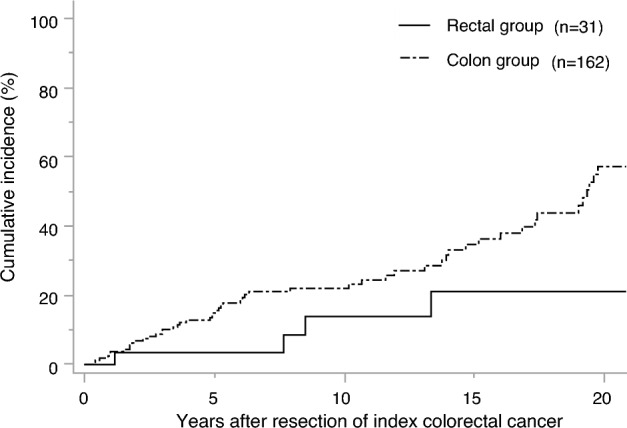
Cumulative risk of metachronous colorectal cancer after surgery for index colorectal cancer by colon or rectum

The cumulative incidence rates at 5, 10, and 20 years in the rectal vs. colon groups were 3.5% vs. 14.9%, 13.9% vs. 22.0%, and 21.1% vs. 57.2%, respectively. The risk of metachronous CRC was significantly lower in the rectal group than in the colon group (*P* = 0.02) (Fig. 1). In the rectal group, metachronous CRC occurred in 6 patients (19.4%), all of whom underwent surgery for metachronous CRC. In the colon group, metachronous CRC occurred in 61 patients (37.7%), of whom 53 (86.7%) underwent surgery, while 8 (13.1%) underwent endoscopic resection.

**Fig. 2:**
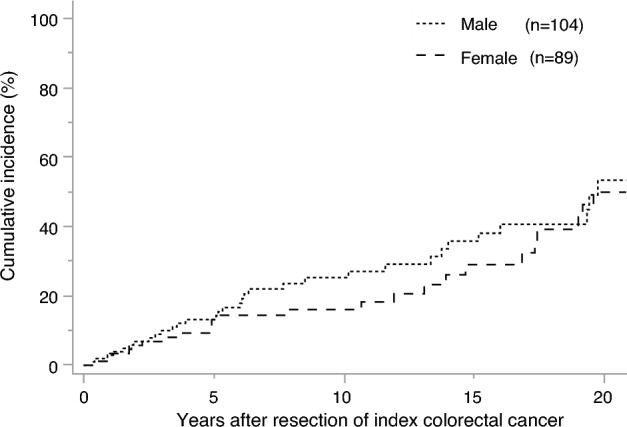
Cumulative risk of metachronous colorectal cancer after surgery for index colorectal cancer by sex

Regarding the cumulative risk of metachronous CRC, the cumulative incidence rates at 5, 10, and 20 years in male and female patients was 13.2% vs. 13.1%, 25.3% vs. 16.1%, and 53.4% vs. 49.9%, respectively. No significant differences were found between the 2 groups (*P* = 0.35) (Fig. 2).

### Causes of death and the OS

**Fig. 3:**
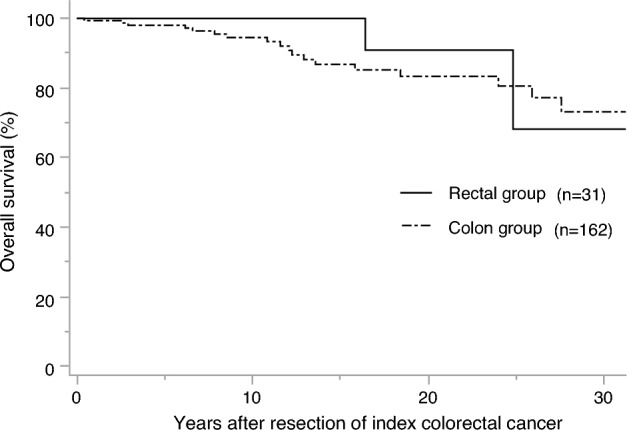
The overall survival after surgery for index colorectal cancer by colon or rectum

**Fig. 4:**
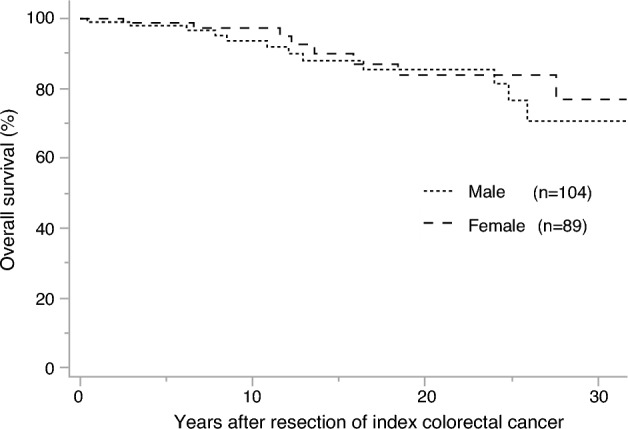
The overall survival after surgery for index colorectal cancer by sex

Twenty-four patients (rectal vs. colon group: 3 vs. 21) died during the observation period, and the causes of death are listed in Table 3. The cause of death in the rectal group was extracolonic LS-associated tumors in 2 patients (66.6%), 1 of which was biliary tract cancer and the other pancreatic cancer. However, CRC was not the cause of death in any of the patients in this group. In the colon group, CRC was the cause of death in 6 patients (28.6%), all of whom were male. LS-associated tumors were the cause of death in 7 patients (33.3%), of whom 5 were female (3 with endometrial carcinoma, 1 with renal pelvic cancer, and 1 with a brain tumor), and 2 male (1with biliary tract cancer and 1 with renal pelvic cancer). The 10-, 20-, and 30-year OS rates in the rectal group were 100.0%, 90.9%, and 68.2%, respectively, and those in the colon group were 94.5%, 83.4%, and 73.2%, respectively. There was no significant difference in the OS between the rectal and colon groups (*P* = 0.23) (Fig. 3). Regarding the OS by sex, the 10-, 20-, and 30-year OS rates for males were 93.6%, 83.9%, and 76.9%, respectively, and those for females were 93.6%, 85.5%, and 70.7%, respectively, with no significant difference observed (*P* = 0.25) (Fig. 4).

**Table 3:** Causes of death after surgery for index colorectal cancer

Cause of death	Colon group (n = 21)			Rectal group (n = 3)		
	All (n = 21)	Males (n = 11)	Females (n = 10)	All (n = 3)	Males (n = 3)	Females (n = 0)
Colorectal cancer	6 (28.6%)	6 (54.5%)	0 (0%)	0 (0%)	0 (0%)	0 (0%)
LS-associated tumors	7 (33.3%)	2 (18.2%)	5 (50.0%)	2 (66.7%)	2 (66.7%)	0 (0%)
LS-unassociated tumors	3 (14.3%)	2 (18.2%)	1 (10.0%)	1 (33.3%)	1 (33.3%)	0 (0%)
Others	1 (4.8%)	0 (0%)	1 (10.0%)	0 (0%)	0 (0%)	0 (0%)
Unknown	4 (19.0%)	1 (9.1%)	3 (30.0%)	0 (0%)	0 (0%)	0 (0%)

*LS* Lynch syndrome

## Discussion

In the present study, the median age at surgery for index CRC and the cumulative incidence rates of metachronous CRC were significantly lower in the rectal group than in the colon group. No significant difference in the OS was found between the two groups. In patients with genetically proven LS, no study has compared the risk and site of metachronous CRC development after index RC surgery with those after index CC surgery. Since index RC accounts for only 10.4%–18.0% of all index CRCs in patients with genetically proven LS [1, 2, 4, 5], there seems to be very little data on metachronous CRC after resection of index RC. In this study, 16.1% of all index CRC in patients with genetically proven LS were RC patients, similar to the findings reported in previous studies. It has been reported that 15%–54% of patients with genetically proven LS or hereditary nonpolyposis colorectal cancer (HNPCC; defined by the clinical criteria only) developed metachronous CRC (Table 4) [9–12].

**Table 4:** Reports on the incidence of metachronous colorectal cancer after surgery for index rectal cancer with LS or HNPCC

	Subjects	Number (genetically proven LS)	Incidence of metachronous CRC	Follow-up time
Möslein et al. [11]	< 55 years old when diagnosed with CRC, having multiple CRCs and/or a family history of cancer	13 (0)	54%	Mean 7.4 years
Lee et al. [9]	Carriers of pathogenic germline variant/Amsterdam criteria/Having a strong clinical story suggestive HNPCC	18 (unknown)	17%	Median 203 months
Kalady et al. [10]	Meeting Amsterdam I or II or Amsterdam-like criteria	33 (0)	15.2%	Median 101.7 months
Win et al. [12]	Carriers of pathogenic germline variant	79 (79)	27%	Median 9 years

*LS* Lynch syndrome, *HNPCC* hereditary nonpolyposis colorectal cancer, *CRC* colorectal cancer

Kalady et al. [10] proposed that TPC with IPAA should be strongly considered as an index RC in patients with HNPCC. However, only 15% of patients with HNPCC after surgery for index RC had metachronous CRC in their study. Among previous studies, only Win et al. [12] targeted genetically proven LS. In that study, metachronous CRC occurred in 21 (27%, over a median of 9 [range, 1–32] years) of 79 patients with LS who underwent anterior or abdominoperineal resection of the index RC, in which the cumulative incidence of metachronous CRC was 19%, 47%, and 69% at 10, 20, and 30 years, respectively. In their report, 76% (16 of 21 patients) of metachronous CRCs were located in the right-sided colon, and none occurred in the remnant rectum. Win et al. [12] also stated that TPC should be considered in patients with LS diagnosed with index rectal cancer. In our study, 83.3% (5 of 6 patients) of metachronous CRC cases that developed after index RC surgery were located in the right-sided colon and none in the remnant rectum, which is consistent with the results reported by Win et al. [12]. However, it has been suggested that the carcinogenic risk of LS may be affected by race, ethnicity, region [13–17], lifestyle, environmental factors [18–22], and genotype [1–3]. Further case series are needed to draw definitive conclusions regarding metachronous CRC after index RC.

Although an analysis of the OS was beyond the scope of the present study, the OS after surgery for CRC was comparable between the rectal and colon groups. Similarly, no significant differences were observed between the male and female patients. Regarding the OS of patients with LS, consideration of the influence of CRC as well as other LS-associated tumors is necessary. Endometrial and ovarian cancers are important LS-associated tumors, particularly in female patients. LS-associated tumors were the most common cause of death in both the groups. Gynecological tumors are the most common cause of death in the colon group. After surgery for index CRC, colonoscopic surveillance and surveillance of extracolonic LS-associated tumors are considered important. Although gynecological tumors are considered particularly important in female patients, whether they contribute to the improvement in the OS remains unclear. Regarding the cause of death, 28.6% of the patients in the colon group had CRC, whereas all deaths in the rectal group were due to extracolonic LS-associated tumors, without any deaths due to CRC. Therefore, extended surgery is unlikely to prolong the survival period of the index RC in patients with LS. Furthermore, extensive surgery, such as IPAA, cannot be recommended as a standard treatment.

In the present study, metachronous CRC occurred in 29.1% of the patients (61 of 162 patients) in the colon group, and 13.1% of the patients had RC (4.9% of all index CC cases). If all patients with index CC had undergone extensive surgery, such as TC with IRA, 62.7% (101 [93 without metachronous CC and 8 with metachronous RC] of 162 patients) would have undergone unnecessary extensive surgery. In the rectal group, metachronous CRC occurred in 19.4% (6 of 31 patients), and all occurred in the colon. If extended surgery, such as TPC with IPAA, had been performed for all index RC cases to avoid metachronous CRC (actually all with metachronous CC), the remaining 80.6% (25 of 31 patients) of index RC patients who did not develop metachronous CRC would have undergone unnecessary extended surgery. Considering the risk and location of metachronous CRC, extended surgery, such as TPC with IPAA, for index RC is less effective than extended surgery, such as TC with IRA, for index CC, from the viewpoint of metachronous CRC reduction and worsening the quality of life after IPAA.

Interestingly, the incidence of metachronous CRC was lower in the rectal group than in the colon group. To our knowledge, no other study has directly compared these cases within the same cohort. In reports from the same study group, Parry et al. [23] reported the incidence of metachronous CRC after index CC surgery, and Win et al. [12] reported the incidence of metachronous CRC after index RC surgery. Although each study was a separate report, the incidence of metachronous CRC after segmental colectomy in 332 index CC cases was 22% (74 cases; median, 9 years). In addition, the cumulative incidences of metachronous CRC were 16%, 41%, and 62% at 10, 20, and 30 years, respectively. The incidence of metachronous CRC after surgical resection (anterior or abdominoperineal resection) in 79 patients with index RC was 27% (21 cases; median, 9 years). Furthermore, the cumulative incidences of metachronous CRC were 19%, 47%, and 69% at 10, 20, and 30 years, respectively. However, the risk of metachronous CRC in patients with index CC previously reported [6, 23–26] was similar to that in the present study. Therefore, more data on metachronous CRC after index RC and the results of the comparison between the two groups must be accumulated in the future before drawing a conclusion.

Regarding the reason for the lower cumulative incidence of metachronous CRC in the rectal group than in the colon group, there were no marked differences in patient characteristics between the two groups other than the age at the onset of index CRC. We evaluated the effects of colonoscopy surveillance after surgery. However, many patients experienced index CRC onset several decades earlier. Therefore, detailed records of colonoscopy surveillance were not obtained. As colonoscopy surveillance within three years after CRC surgery has been recommended in patients with LS [27–29], we reviewed whether or not colonoscopy had been performed even “within three years.” There was no significant difference in the performance of endoscopy surveillance within three years after resection of the index CRC between the colon and rectal groups. Surgeons and oncologists in Japan seem to have recognized the importance of postoperative colonoscopy surveillance even before the publication of the guideline [30]. Therefore, postoperative colonoscopy surveillance was performed satisfactorily, regardless of the index CRC site, and the cumulative incidence of metachronous CRC was unaffected. The further accumulation of cases is necessary to reach a consensus on whether or not there is a biologically different background between the index CC and index RC in patients with LS.

Several limitations associated with the present study warrant mention. First, this was a retrospective study, the sample size was relatively small, and the follow-up period after index CRC surgery was too short to evaluate the lifetime risk of metachronous CRC. In particular, the frequency of index RC in patients with LS was as low as previously reported. Second, we were unable to examine the detailed interval of colonoscopy surveillance after surgery for the index CRC and the quality of colonoscopy management. In addition, no assessment was performed for any causative gene. Despite these limitations, we believe that this study holds value in its reporting of the incidence and sites of metachronous CRC in patients with LS with index RC and index CC. Thus, this report will help consider the optimal surgical treatment for index RC.

To our knowledge, there have been no other reports on index RC and metachronous CRC in patients with LS in East Asia, including Japan. In conclusion, we cannot recommend extended surgery, such as TPC with IPAA, as the standard surgical treatment for index RC in patients with LS. Careful colonoscopy surveillance for metachronous CRC and surveillance for extracolonic LS-associated tumors might be important, although further research with a larger cohort is needed to achieve definitive conclusions.
